# The Ten-Group Robson Classification: A Single Centre Approach Identifying Strategies to Optimise Caesarean Section Rates

**DOI:** 10.1155/2017/5648938

**Published:** 2017-01-10

**Authors:** Keisuke Tanaka, Kassam Mahomed

**Affiliations:** ^1^Department of Obstetrics and Gynaecology, Ipswich Hospital, Ipswich, QLD, Australia; ^2^Ipswich Hospital and University of Queensland, Ipswich, QLD, Australia

## Abstract

Caesarean section (CS) rates have been increasing worldwide and have caused concerns. For meaningful comparisons to be made World Health Organization recommends the use of the Ten-Group Robson classification as the global standard for assessing CS rates. 2625 women who birthed over a 12-month period were analysed using this classification. Women with previous CS (group 5) comprised 10.9% of the overall 23.5% CS rate. Women with one previous CS who did not attempt VBAC contributed 5.3% of the overall 23.5% CS rate. Second largest contributor was singleton nulliparous women with cephalic presentation at term (5.1% of the total 23.5%). Induction of labour was associated with higher CS rate (groups 1 and 3) (24.5% versus 11.9% and 6.2% versus 2.6%, resp.). For postdates IOL we recommend a gatekeeper booking system to minimise these being performed <41 weeks. We suggest setting up dedicated VBAC clinic to support for women with one previous CS. Furthermore review of definition of failure to progress in labour not only may lower CS rates in groups 1 and 2a but also would reduce the size of group 5 in the future.

## 1. Introduction

Rising caesarean section (CS) rate is of worldwide concern particularly in well-resourced countries [1]. The rate has increased from 23.3% in 2000 to 33% in 2013 in Australia [2, 3]. It has been reported that rates higher than 9–16% are not associated with decreases in maternal and neonatal mortality [4, 5]. There is growing concern about the higher incidence of long-term complications following one or more CS such as placenta accreta, retained placenta, and uterine rupture with possible need for peripartum hysterectomy [6–8]. Another concern is the varying rates of CS among member hospitals of Women's Healthcare Australasia (WHA), ranging from 18% to 37% [9]. This has been assumed to be due to a variation in the obstetric populations.

A recent systematic review of 27 different classifications [10] suggested that the Ten-Group Robson classification of caesarean sections [11] might allow us to look at CS rates in specific groups to help identify possible reasons for this variation. Women who give birth are categorised into 10 groups based on their basic obstetric characteristics of parity, previous CS, gestational age, mode of onset of labour, fetal presentation, and number of fetuses. These groups are structured in such a way that they are mutually exclusive and totally inclusive. The Ten-Group Robson classification has been praised for its simplicity, robustness, reproducibility, and flexibility [12] and has been recommended for both the monitoring rates over time as well as between facilities by both WHO in 2014 and FIGO in 2016 [13, 14].

Various modifications or subdivisions to the original ten groups have been suggested such as having subdivisions based of the mode of onset of labour [15].

Prior to introducing interventions to address the rising CS rates we have classified all women who gave birth over a 12 months period from January to December 2015, using the Ten-Group Robson classification with subdivisions based on onset of labour.

## 2. Materials and Methods

The study population included all live births and stillbirths of at least 400-gram birth weight or at least 20-week gestation at Ipswich Hospital, Queensland, Australia, during the period January–December 2015. Ipswich Hospital is a secondary referral University teaching hospital, supported by 24-hour theatre, anaesthetic, and paediatric services with a special care nursery equipped to care for neonates from 32-week gestation.

Data were extracted from the National Perinatal Data Collection (NPDC), an Australian population-based cross-sectional data collection of pregnancy and childbirth and cross checked with the birth suite register to ensure that no CS were missed. Medical records were reviewed for missing or for verifying information. Overall CS rate, relative size of each group, CS rate, and relative contribution of each group to the overall CS rate were calculated.

As this review conforms to the standards established by National Health and Medical Research Council for ethical quality review [16], ethics approval was not sought.

## 3. Results

2625 women gave birth to 2663 babies at Ipswich Hospital in 2015. CS was performed in 618 women resulting in an overall CS rate of 23.5%. Women in each of the ten groups are shown in Table 1. The table also shows the CS rate in each of these 10 groups as well as the contribution of each group to the overall CS rate of 23.5%.

The largest contributor to the overall CS rate was women with previous CS (group 5), 10.9% of the overall 23.5%. CS rate in this group was 76.5% (287 out of 375 women). 224 out of 287 women (78.0%) had the CS performed prior to onset of labour (group 5c). Of the 250 women who had had one previous CS in group 5, altogether 110 (44%) attempted VBAC. A significant number of these would have been multiparous women with previous vaginal births. We do not have the number of women with CS in first pregnancy who attempted a VBAC in the second pregnancy.

The second largest contributor was groups 1 and 2 combined, the singleton nulliparous women with cephalic presentation at term. This group that comprised 28.9% of the total population had an overall CS rate of 17.9% and accounted for 5.1% of the total CS rate of 23.5%. The prelabour CS rate in this group (group 2b/group 1 + group 2) was 1.7% (13 out of 759).

## 4. Discussion

We present our data to encourage other obstetric units to adopt this classification that is simple to incorporate into the routine perinatal data collection system. CS rates for each of the 10 groups can then become more meaningful and rates for each group can then be compared with other obstetric units. Secondly, by identifying groups that contribute most to the CS rate in our unit, as we believe they would be similar in other units as well, quality improvement activity could be initiated to modify the CS rate in a particular group.

The low CS rates, 11.9% in nulliparous women in spontaneous labour, 16.6% in multiparous women, and only 4.7% of women birthing before 37 weeks, indicate that we are dealing with a relatively low risk population.

Group 5 (previous CS, singleton cephalic, ≥37 weeks) was the largest contributor to the overall CS rate (10.9% of the total 23.5%) mostly due to women having CS prior to labour (group 5c). It is a common practice to recommend an elective repeat CS to women with more than one previous CS; we did not have detailed information on women with one previous CS who attempted vaginal birth. We did note however that 56% of women with one previous CS elected to have a repeat CS for whatever reason and that 44% elected to attempt vaginal birth after CS. We do not have the percentage of women with CS in their first pregnancy who had planned to have a VBAC. Even though vaginal birth after one CS has been advocated as a safe option [17–19], the number of women who attempt VBAC has declined over recent years due to fear of uterine rupture [20, 21]. Some centres have set up dedicated VBAC clinics to assist women to make an informed choice with use of decision aids and have noted an increase in the number of women electing to have a VBAC [22, 23].

Women in group 1 who went into spontaneous labour had a CS rate of 11.9% as opposed to similar women whose labour was induced (group 2a) who had a CS rate of 24.5%. Number of women whose labour is being induced is growing [11]. However within this group the commonest indication for induction is “postdates.” We recently reviewed all births in the state of Queensland in Australia where it is standard policy to induce labour for postdates after 41 completed weeks [24]. It was of concern that as many as 36% of women in this group were induced at between 40^0^ and 40^6^ weeks. Any reduction in CS in this group would affect the CS rate in the total group of nulliparous women with a potential for vaginal birth and would also reduce number of women in group 5 in the years to come [25].

We believe that obstetric units should critically address two issues. The first is that we need to be as evidence based as possible in recommending an IOL [26–28]. Limiting IOL for which there is no clear indication, especially those with an unfavourable cervix, would have a significant effect of the CS rate. The two recent reviews that concluded that IOL is not associated with an increase in CS rate [29, 30] are likely to encourage clinicians to be more liberal in recommending IOL, despite numerous weaknesses in many of the randomised controlled trials included in the reviews. Our plan in the first instance is to modify our procedure of induction for “postdates” to adhere to a policy of induction after 41 completed weeks with bookings being made by a “gate keeper” to ensure that routine inductions are not performed before then.

The second issue is to address one of the two commonest indications for a primary CS; failure to progress and fetal heart rate concern. Increasing maternal age, maternal and fetal weight, common obstetric interventions such as induction, epidural analgesia, and oxytocin use may have altered what would be normal progress of labour. A large study on singleton, cephalic term pregnancies in spontaneous labour concluded that active labour with cervical dilatation of 0.5 to 1 cm per hour only begins after 6 cm dilatation and it may take longer than currently expected normal time frame for many women to reach 6 cm cervical dilatation [31]. It is possible that some women may be having a CS for failure to progress when they have not even begun to be in active labour [32]. We aim to review on a daily basis all emergency CS in the previous 24 hours to critically evaluate this as an indication.

Increasing CS rate among women with breech presentation is a common phenomenon particularly since the publication of the term breech trial [33–35], and our hospital is not an exception. Groups 6 and 7 consist of women with breech presentation and showed high CS rates. Despite the criticisms of the term breech trial [36–38], many hospitals including ours have been reluctant to offer vaginal breech birth. Even though this group is relatively small, we should however be more proactive in offering external cephalic version to all eligible women with breech presentation and consider offering vaginal breech delivery with clear guidelines to suitable women. Use of Ten-Group Robson classification will eventually allow us to directly or indirectly compare specific subgroups of our obstetric population.

## 5. Conclusions

The Ten-Group Robson classification is only a starting point but it is important to have a common starting point. It enables us not only to understand the different obstetric groupings but also to monitor changes over time at one facility as well as being able to compare practices between facilities. Having implemented the Robson classification and identified groups which contributed the most to the overall CS rate, we hope to report at a later date any effect this may produce after introducing the two interventions mentioned above.

## Figures and Tables

**Table 1:** Rate of Caesarean section by the Ten-Group Robson classification.

	Relative size of groups (% of total number of births)	CS rate in each group (% of number of women in each group)	Contribution made by each group to the overall CS rate %
(1) Nulliparous, single cephalic, ≥37 weeks, spontaneous labour	**18.6%**	**58/489 (11.9%)**	**2.2%**
(2) Nulliparous, single cephalic, ≥37 weeks	**10.3%**	**76/270 (28.1%)**	**2.9%**
(a) Induced	9.8%	63/257 (24.5%)	2.4%
(b) CS before labour	0.5%	13/13 (100%)	0.5%
(3) Multiparous, single cephalic, ≥37 weeks, spontaneous labour	**34.4%**	**26/902 (2.9%)**	**1.0%**
(4) Multiparous, single cephalic, ≥37 weeks	**13.8%**	**60/361 (16.6%)**	**2.3%**
(a) Induced	12.2%	20/321 (6.2%)	0.8%
(b) CS before labour	1.5%	40/40 (100%)	1.5%
(5) Previous CS, singleton cephalic, ≥37 weeks	**14.3%**	**287/375 (76.5%)**	**10.9%**
(a) Spontaneous labour	4.5%	54/119 (45.4%)	2.1%
(b) Induced	1.2%	9/32 (28.1%)	0.3%
(c) CS before labour	8.5%	224/224 (100%)	8.5%
(6) All nulliparous breeches	**0.9%**	**21/23 (91.3%)**	**0.8%**
(a) Spontaneous labour		5/6	
(b) Induced		0/1	
(c) CS before labour		16/16	
(7) All multiparous breeches^*∗*^	**1.1%**	**26/29 (89.7%)**	**1.0%**
(a) Spontaneous labour		9/11	
(b) Induced		0/1	
(c) CS before labour		17/17	
(8) All multiple pregnancies^*∗*^	**1.4%**	**20/38 (52.6%)**	**0.8%**
(a) Spontaneous labour		7/15	
(b) Induced		3/13	
(c) CS before labour		10/10	
(9) All abnormal lies^*∗*^	**0.5%**	**14/14 (100%)**	**0.5%**
(a) Spontaneous labour		5/5	
(b) Induced		4/4	
(c) CS before labour		5/5	
(10) All singleton cephalic, ≤36 weeks^*∗*^	**4.7%**	**30/124 (24.2%)**	**1.1%**
(a) Spontaneous labour		9/67	
(b) Induced		3/39	
(c) CS before labour		18/18	

Total	**100%**	**618/2685**	**23.5%**

^*∗*^Groups 7–10 include women with previous CS.
